# Use of Genotyping to Disprove a Presumed Outbreak of *Mycobacterium tuberculosis* — Los Angeles County, 2013–2014

**Published:** 2014-10-10

**Authors:** Brian J. Baker, Shameer Poonja, Myrna Mesrobian, Anna Lai, Steven Hwang

**Affiliations:** 1 Division of Tuberculosis Elimination, National Center for HIV/AIDS, Viral Hepatitis, STD, and TB Prevention, CDC; 2 Los Angeles County Department of Public Health

In early 2013, the Los Angeles County Department of Public Health learned of two patients diagnosed with tuberculosis (TB) who had received care at the same outpatient health care facility (facility A). Facility A is a center for infusions of chemotherapeutic and biologic agents and serves a large number of immunocompromised persons who were potentially exposed to infectious TB. If infected, immunocompromised persons are at elevated risk for progression to TB disease (1). The two patients (patient A and patient B) both had pulmonary TB, with acid-fast bacilli found on sputum-smear microscopy, and had visited facility A multiple times during their infectious periods. Despite initial concerns that these two cases could be the result of person-to-person transmission at facility A, genotyping of the *Mycobacterium tuberculosis* isolates from these two patients showed that they were infected with unrelated strains.

During the investigation surrounding the first two patients, two additional patients (patient C with TB adenitis and patient D with pulmonary TB) were found to have been present at facility A during the infectious period of patient B. Three of the four patients were born in countries with a high TB prevalence, and all four patients had significant comorbidities that promote the progression of *M. tuberculosis* infection to TB disease (e.g., malignancy, malnourishment, and diabetes mellitus). Of the 281 persons potentially exposed to either patient A or patient B in the waiting area or in one of the infusion rooms at facility A, 261 were initially evaluated. Evaluation of facility contacts was difficult and resource-intensive, because many patients at the facility had abnormal chest radiographs due to malignancy or radiation therapy, and health care providers set low thresholds for initiating evaluations of TB disease for these patients. Because of the epidemiologic connections between patients B, C, and D and the high prevalence of immunocompromising conditions among exposed persons, further expansion of the investigation was considered to address a presumed outbreak. However, while expansion was under consideration, genotyping results were received for the mycobacterial isolates from patients C and D; these results differed from each other and from the isolates from patients A and B, conclusively demonstrating that the four cases were unrelated (2).

Previous studies have demonstrated the value of genotyping to identify previously unrecognized outbreaks (including those across multiple jurisdictions) and to prioritize resources for public health action (3,4). In this instance, timely genotyping demonstrated that a presumed outbreak of TB was actually a series of unrelated cases, thereby allowing the Los Angeles County Department of Public Health to avoid expanded testing and save valuable resources. In settings with large numbers of foreign-born persons with risk factors for progression to TB disease (as was the case with facility A), coincidental groups of TB cases might be found given the expected high incidence of TB disease in these populations. Examples such as the one described in this report reflect the changing nature of TB epidemiology in Los Angeles County and the United States. Almost two thirds of reported TB cases in the United States are in foreign-born persons (5), and three fourths of the cases among foreign-born persons are likely the result of reactivation of latent TB infection, rather than person-to-person transmission in the United States (6).

As part of its investigation, Los Angeles County Department of Public Health recommended that future patients at facility A be routinely tested for *M. tuberculosis* infection, with treatment of persons found to have latent TB infection. Six months after the diagnosis of patient D, a fifth patient who had also received care at facility A was diagnosed with pulmonary TB; genotyping demonstrated that the fifth case was unrelated to the other four cases. Possible outbreaks of TB require an urgent public health response to interrupt further transmission; timely universal genotyping can ensure informed and efficient use of limited public health resources.
